# The effects of laser procedure in symptomatic patients with haemorrhoids: A systematic review

**DOI:** 10.3389/fsurg.2022.1050515

**Published:** 2022-12-12

**Authors:** Gonçalo Torrinha, Tatiana Gonçalves, Maria Sousa, Gerrit Högemann, André Goulart, Alexandre Fernandes Carvalho, Pedro Leão

**Affiliations:** ^1^ Life and Health Sciences Research Institute (ICVS), Medical School, University of Minho, Braga, Portugal; ^2^ General Surgery Department, Hospital Privado de Braga, Grupo Trofa Saúde, Braga, Portugal; ^3^ General Surgery Department, Städtisches Klinikum Karlsruhe, Karlsruhe, Germany; ^4^ ICVS/3B's – PT – Surgical Science Department, Government Associate Laboratory, Braga/Guimarães, Portugal

**Keywords:** haemorrhoids, diode laser, haemorrhoidectomy, haemorrhoidplasty, HeLP

## Abstract

**Purpose:**

Haemorrhoids are normal structures in the human body, only seen as pathological when symptomatic. Nowadays, new techniques have surfaced using a diode laser which, after locating the target arteries, blocks the blood flow while hitting and shrinking the local mucosa/submucosa at a depth of 4 mm. Our work aimed to give a broad view over this new technique and its consequences in the post-operative follow-up with a systematic review.

**Methods:**

EMBASE and MEDLINE databases were consulted, retrieving clinical trials, which mentioned the use of 980 nm diode laser on the treatment of haemorrhoids.

**Results:**

Ten clinical trials analyzing the post-operative effects of laser haemorrhoidectomy were selected, including 2 randomized controlled clinical trials and 1 controlled clinical trial. The overall quality of the trials was low, indicating a high risk of bias.

**Conclusion:**

The laser haemorrhoidectomy procedure revealed a high therapeutic potential, considering the reduced number of postoperative complaints (bleeding/pain), the high symptom resolution and the reduced recurrence, albeit the high heterogeneity between the studies in terms of reported results. Future investigations with higher quality and controlled double-blinded studies obtaining better-categorized results should be conducted in order to better evaluate this procedure and compare it to the current paradigm.

## Introduction

### Description of the condition

Haemorrhoids are normal structures of the human body (1–3). They are usually classified by their location: internal (originates above the dentate line and covered by anal mucosa) and external (originates below the dentate line and covered by anoderm) (2, 4). The internal haemorrhoidal plexus presents itself as anal cushions or sinusoids and consists of arterioles, venules and arteriovenular anastomoses (5).

Generally, haemorrhoids are viewed as a disease when they become symptomatic (6). The most common presentation of hemorrhoidal disease is painless rectal bleeding during defection (3, 6). Pain in patients with haemorrhoids is more likely due to anal fissures and anorectal abscesses (7).

The pathophysiology of internal haemorrhoids is still not fully understood. However, one theory postulates that a chronic rise in intra-abdominal pressure in combination with the absence of valves within rectal veins, can limit venous drainage from sinusoids during defecation, resulting in abnormal dilatation of the sinusoids and, in turn, bleeding due to bursting and/or mucosal damage (2, 8). The Goligher classification is commonly used to grade the severity of haemorrhoids and consequently indicates the modality of surgical treatment (9). Grade I corresponds to non-prolapsing haemorrhoids and grade II to prolapsing haemorrhoids on defecation with spontaneous reduction (10). In advanced stages, the additional disintegration of conjoined longitudinal muscle results in their remaining permanently outside the anus, either manually reversible (3rd degree) or nonreversible (4th degree) (11, 12).

### Description of the intervention

The haemorrhoidal laser procedure (HeLP) uses a diode laser, delivered at 980 nm of wavelength (13 W: 5 pulsed shots of 1.2 s each with 0.6 s pause), acting selectively on haemoglobin and causing the closure of the superior hemorrhoidal arteries. No general anesthesia is required for this procedure and, if requested by the patient, analgesic drugs can be administered intraoperatively. A Doppler-transducer (20 Mhz probe of 3 mm diameter) can be used to help identify the terminal branches of the superior hemorrhoidal arteries approximately 3 cm proximal to the dentate line. This device is inserted into the rectum with the patient in lithotomy position, being substituted by the laser after identification of the target artery (13).

### How the intervention might work

The laser cuts off the blood supply to the haemorrhoids and causes shrinkage of the mucosa and submucosa, to a depth of 4 mm (14).

### Why this review is important

So far, Milligan-Morgan haemorrhoidectomy is the most commonly used technique in Europe (15). However, this technique is invasive and may lead to severe postoperative pain. An ideal procedure for the treatment of haemorrhoids should have the most symptom resolution paired with minimal postoperative pain and complications, as well as demonstrate less recurrence. The procedure should be cheap and cost-effective too.

With this review we aimed to analyze the effects of the laser procedure in terms of post-operative complications and morbidities and access the therapeutic potential of this procedure.

## Methods

This review was performed according to the Preferred Reporting Items for Systematic Reviews and Meta-Analyses (PRISMA) norms (16, 17).

### Eligibility criteria

All studies regarding the use of diode lasers with a wavelength of 980 nm on the treatment of haemorrhoids were considered.

Only trials conducted in humans, published in English, reporting original results were selected. Conference abstracts, reviews, commentaries, case reports and book chapters were excluded.

### Information sources

Studies were identified by searching the electronic databases MEDLINE and EMBASE. This search was last conducted by the authors on 18th of June 2019.

### Search

The following setup of search terms was used for MEDLINE: “(‘haemorrhoidectomy’[MeSH Terms] OR ‘haemorrhoidectomy’[All Fields]) AND (‘lasers’[MeSH Terms] OR ‘lasers’[All Fields] OR ‘laser’[All Fields])”; “(‘lasers’[MeSH Terms] OR ‘lasers’[All Fields] OR‘laser’[All Fields]) AND (‘haemorrhoids’[All Fields] OR ‘haemorrhoids’[MeSH Terms] OR ‘haemorrhoids’[All Fields])”.

The following setups of search terms were used for EMBASE: “‘hemorrhoids laser’ OR ((‘hemorrhoids’/exp OR hemorrhoids) AND (‘laser’/exp OR laser))” and “‘hemorrhoidectomy laser’ OR ((‘hemorrhoidectomy’/exp OR hemor- rhoidectomy) AND (‘laser’/exp OR laser))”.

### Study selection

The authors performed an eligibility assessment. In case of questionable eligibility, the results were discussed among all authors. All trials were included, regardless of the existence and type of a comparative group. The primary outcome measure was the impact of the laser on pain and bleeding on post-operative follow-up. The secondaries outcomes were: number of arteries affected by laser, mean time of surgery, time until discharge, resolution and recurrence of symptoms after surgery. Articles including participants with previous surgical treatments for haemorrhoids and concomitant anorectal disorders were excluded. Articles that did not use 980 nm diode lasers as a therapeutic approach or used the laser pared with other surgical procedures were also excluded.

### Data collection process

We developed a data extraction sheet with the descripted data of each report, adding new parameters throughout the analysis as soon new data was found. All data extracted by the authors was reviewed twice to avoid errors. In cases of uncertain validity, the results were discussed among all authors. Studies from the same research group or group of authors were carefully analyzed to avoid double counting the same data.

### Data items

From each study, we extracted the following data items: (1) participant groups (country, sample size, mean age and gender ratio); (2) disorder (haemorrhoid degree); (3) laser procedure (type of anaesthesia/analgesia, number of arteries affected by laser, mean time of surgery and if the laser procedure was or not Doppler-guided) and (4) main outcome measures (bleeding, pain, time until discharge, resolution and recurrence of symptoms after surgery).

### Risk of bias in individual studies

To ascertain the risk of bias of the eligible studies, the authors determined the quality of each study using the critical appraisal skills programme (CASP) checklist for randomized controlled trials (18).

In the case of trials, which did not have a comparison/control group, the methodological quality assessment was established by using the Quality Assessment Tool for Before-After Studies with No Control Group (National Heart Lung and Blood Institute).

### Synthesis of results

In order to extract data regarding the outcome variable bleeding, we focused our attention on the occurrence of bleeding after the surgery, only extracting the number of patients with bleeding after laser procedure up to 1 month after surgery.

Concerning the outcome variable “pain”, we extracted “early post-operative pain” data expressed using a visual analogue scale (VAS), as well as the maximum percentage of patients with pain up to 12 months of follow-up. The mean and range values for early post-operative pain data was selected and pulled out according to the following time parameters: first 24 h, 1–3 days and 4–14 days. If the mean value wasn't available, the proportion of patients per VAS score was retrieved; if the range value wasn't available, standard deviation was extracted instead. Each time the percentage of patients with pain was presented as divided between “pain during defecation” and “pain at rest”—we considered “pain” as the sum of these two variables.

Other outcome variables observed were the time until discharge (mean hours and standard deviation, when available), the percentage of patients with complete resolution of symptoms and the percentage of patients with recurrence after 12 months of follow-up.

## Results

### Study selection

**Figure 1:**
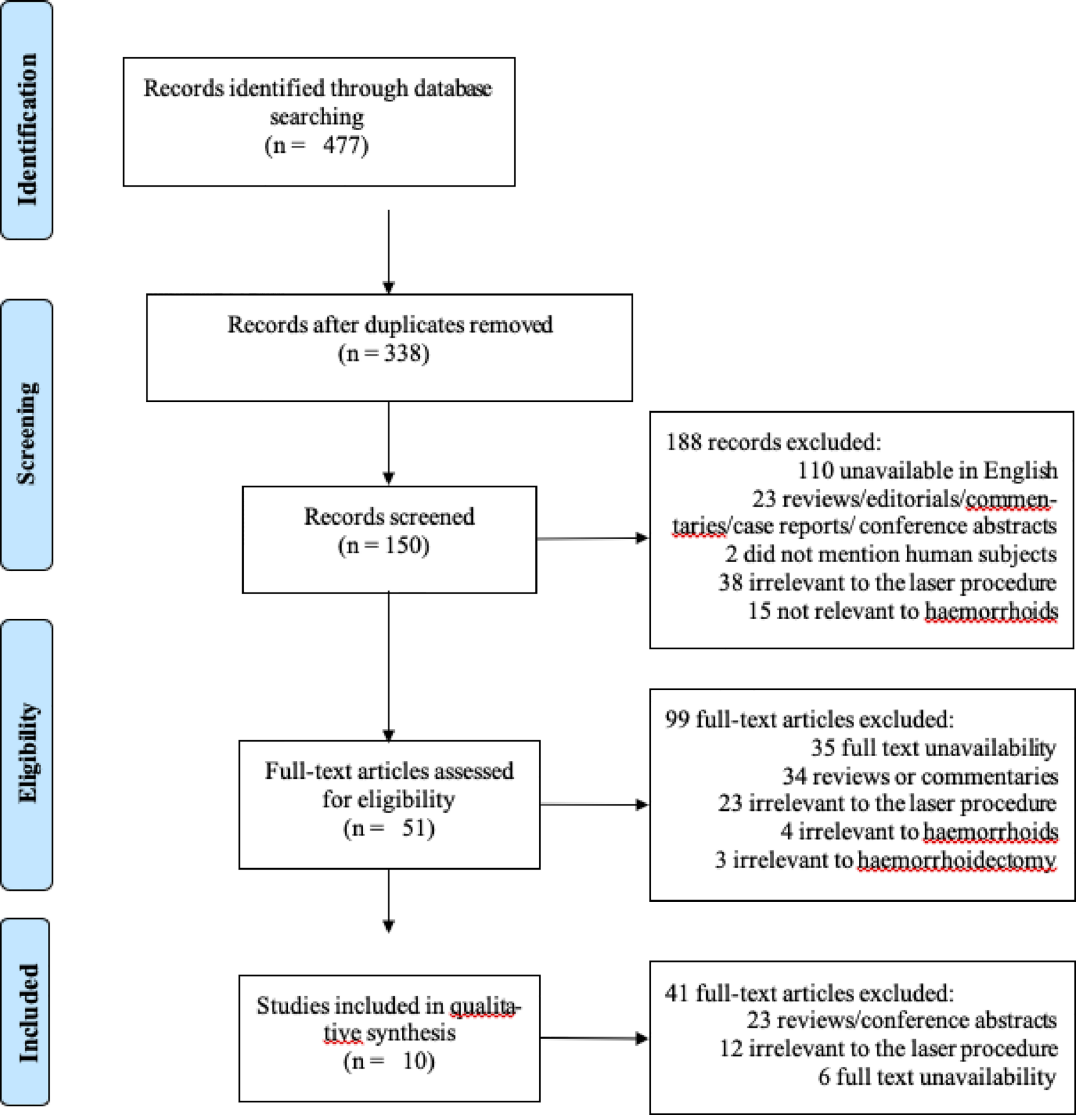
Summary of data collection process.

Figure 1 shows the flow diagram representative of the process of study selection. We retrieved 338 potentially relevant reports from our electronic searches. From these, 51 studies were elected to be included in the review after reading the abstract and removing duplicates. From those, 6 articles were discarded due to full text unavailability, as well as 2 reviews and 21 conference abstracts. Twelve studies did not meet the inclusion criteria: no use 980 nm diode lasers (*n* = 1). no description of the employed laser (*n* = 7) and no use of diode lasers at all (*n* = 4).

### Study characteristics

All studies included in the systematic review (*n* = 10) involved the hemorrhoidal laser procedure and were published between 2009 and 2018.

We analyzed three randomized controlled trials; one was performed in Italy (19), one in Kosovo (20), and the other in Iran (21). Two prospective clinical trials were included in our search: one multicentric (22) and another not multicentric (23), both conducted in Italy. Five remaining studies were all clinical trials performed in Italy (13, 24, 25), Israel (15) and Iran (26). Table 1 shows a summary of the studies included in the systematic review. The mean age of the participants for each study ranges between 41.5 and 47.5 years of age [2 articles didn't report mean age (19, 26)]. The sample size ranges between 20 and 341; predominance in male subjects can be observed in most trials, except for two reports (13, 25).

**Table 1:** Summary of demographic and clinical information of the studies included for systematic reviewing.

Study	Country	Sample size	Gender (M | F)	Mean age (years)	Stage disease (I | II | III | IV degree)
Giamundo et al., 2018	Italy	284	183 | 101	47.5 (17–77)	5 | 174 | 101 | 4
Giamundo et al., 2010	Italy	30	16 | 14	47 (25–70)	0 | 14 | 16 | 0
Giamundo et al., 2011	Italy	30	13 | 17	47 (24–70)	0 | 20 | 10 | 0
Crea et al., 2014	Italy	97	53 | 44	47 (36–59)	0 | 51 | 46 | 0
De Nardi et al., 2016	Italy	51	36 | 15	44 (18–70)	0 | 29 | 22 | 0
Ram et al., 2018	Israel	62	41 | 21	41.5 (24–67)	0 | 18 | 44 | 0
Naderan et al., 2017	Iran	30	13 | 17	43.7 ± 13.7	0 | 13 | 17 | 0
Jahanshahi et al., 2012	Iran	341	219 | 122	(21–100)	0 | 127 | 34 | 2
Maloku et al., 2014	Kosovo	20	11| 9	47 (24–70)	0 | 0 | 20 | 0
Salfi, 2009	Italy	200	72 | 128	21–81	200 (II and III)

Table 2 shows the inclusion and exclusion criteria used by each study. All studies included symptomatic patients, II/III degree, minimal prolapse and failure of conservative treatment. Five full-texts excluded previous surgical treatment, severe prolapse, previous surgical anastomosis less than 3 cm from the dentate line, anal stenosis, fissures and fistulas, and patients under treatment with anticoagulants (15, 19, 21–23). One study included patients taking Low Molecular Weight Heparin (LMWH) and patients with first and fourth degree hemorrhoids (24), and one study included patients with fourth degree hemorrhoids (26). Some articles didn't specify a great number of criteria (20, 25, 26). However, since they followed the HeLP protocol (13) which stated specific guidelines, the assumption was made that inclusion/exclusion criteria were similar.

**Table 2:** Summary of the inclusion and exclusion criteria of the studies included for systematic reviewing.

Study	Inclusion criteria	Exclusion criteria
Giamundo et al., 2018	• Symptomatic patients Failure of conservative treatment • Low or moderate prolapse • Recurrent bleeding and acute symptoms after failure of previous surgical treatments • Patients using LMWH^a^	• None
Giamundo et al., 2010	• Symptomatic patients • Low or moderate prolapse • II- and III-degree hemorrhoids	• IV degree
Giamundo et al., 2011	• Symptomatic patients Minimal prolapse • II- and III-degree hemorrhoids 18–70 years	• Previous surgical treatments for hemorrhoids • Inflammatory bowel disease Fecal incontinence • Obstructed defecation syndrome resistant to medical therapy • Previous surgical anastomosis less than 3 cm from the dentate line • Anal stenosis Fissures or fistulas • Current treatment with anticoagulant medications • Irritable bowel syndrome with severe constipation or diarrhea symptoms
Crea et al., 2014	• II and III degree Low or moderate prolapse	• IV degree • III degree with severe prolapse • <18 and >75 years • Previous surgery for hemorrhoids • Inflammatory bowel disease Obstruction defecation syndrome • Previous surgical anastomosis lower than 5 cm from the dentate line • Anal stenosis Fissure or fistulas • Current treatment with anticoagulant • Thrombosis of hemorrhoidal cushions • Fecal incontinence (Wexner >7)
De Nardi et al., 2016	• Failure of conservative treatment Minimal prolapse • II and III degree	• IV degree Severe prolapse • Previous surgical treatment for hemorrhoids • Inflammatory bowel disease Previous rectal anastomosis lower than 5 cm from the dentate line Anal stenosis • Fissure or fistula • Current treatment with anticoagulant Thrombosis of hemorrhoidal cushions Fecal incontinence
Ram et al., 2018	• Symptomatic patients Failure of conservative treatment • Minimal prolapsed II and III degree	• IV degree Severe prolapse • Previous rectal anastomosis lower than 5 cm from the dentate line • Anal stenosis Fissure or fistula • Thrombosis of hemorrhoidal cushions
Naderan et al., 2017	• Symptomatic patients Failure of conservative treatment • II and III degree	• Previous surgical treatment for hemor- rhoids • Inflammatory bowel disease Fissure • Thrombosis of hemorrhoidal cushions Substance abuse • Liver cirrhosis Kidney disfunction
Jahanshahi et al., 2012	• II, III, IV degree and mixed type of hemorrhoids	• Fissure or fistula
Maloku et al., 2014	• Moderate prolapse III degree	• IV degree and prolapse
Salfi, 2009	• II and III degree	• None

^a^ LMWH, low molecular weight heparin.

Table 3 summarizes the surgical procedure information of each study. Out of the 10 studies, two didn't use any anesthesia throughout the procedure (19, 25), two refer some use of topical anesthesia (22, 24), and one mentioned the use of general anesthesia in all patients (21). The overall number of arteries that have undergone the procedure ranged between 8 and 15. The mean surgery duration time ranged between 9.5 and 33.1 min. Almost all studies performed the doppler-guided laser procedure, except for three: Jahanshashi et al., 2012 (26) and Maloku et al., 2014 (20) and Naderan et al., 2017 (21).

**Table 3:** Summary of the surgical procedure information of each study by employed anaesthetics, number of arteries implied, procedure time and Doppler assistance.

Study	Anesthesia/Analgesic	Number of arteries	Mean time of surgery (minutes)	Doppler- guided laser procedure
Giamundo et al., 2018	• Lidocaine/pilocarpine 5% cream in 246 cases (86.7%) • Light sedation [intravenous • (IV) midazolam, 2 mg] was induced in 34 patients (12%) • Local or spinal anesthesia was used for four patients (1.3%)	12	15.5 (7–31)	Yes
Giamundo et al., 2010	In three patients, a minor analgesic drug (ketorolac 40 mg and/or paracetamol 500 mg) was administered during the operation at the patient's request	10.8 ± 1.2 (8–12)	9.5 ± 2.3	Yes
Giamundo et al., 2011	No patients required anesthesia during the procedures	(8–12)	10 median (7.8–11.2)	Yes
Crea et al., 2014	All operations were carried out with- out general or local anesthesia; Nonsteroidal anti-inflammatory drugs, usually ketorolac, were administered intravenously only on de- mand	10 (5–13)	18 median (12–40)	Yes
De Nardi et al., 2016	The operations were carried out un- der topical anesthesia (EMLA: oint- ment: lidocaine 2.5% and prilocaine 2.5%)	13 median (10–15)	21.29 ± 5.6	Yes
Ram et al., 2018	Fifty-eight laser procedures were performed under sedation, and 4 without any anesthesia	(8–12)	16.6 ± 3.7 (II degree) 20.8 ± 2.5 (III degree)	Yes
Naderan et al., 2017	All procedures were performed under general anesthesia with the same anesthesia protocol	–	33.1 ± 7.3	No
Jahanshahi et al., 2012	General or spinal anesthesia	–	10 (5–15)	No
Maloku et al., 2014	-	–	15.94 ± 3.5	No
Salfi, 2009	Anesthetic or analgesic treatment was not necessary	–	15	Yes

### Risk of bias within studies

Tables 4, 5 compile the information regarding the quality of the studies included in the systematic review.

**Table 4:** Summary of the results of the CASP appraisal list.

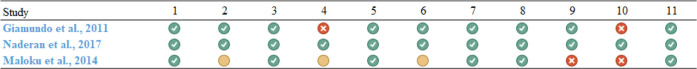	
Evaluated parameters
1	Did the trail address a clearly focused issue?
2	Was the assignment of patients to treatments randomized?
3	Were all patients who entered the trial properly accounted for at its conclusion?
4	Were patients, healthcare workers and study personell “blind” to the treatment?
5	Were the groups similar at the start of the trial?
6	Aside from the experimental intervention, were the groups treated equally?
7	How large was the treatment's effect?
8	How precise was the estimate of the treatment effect?
9	Can the results be applied to the local population, or in your context?
10	Were all clinically important outcomes considered?
11	Are the benefits worth the harms and costs?

**Table 5:** Quality assessment tool for before-after studies with no control group (national heart lung and blood institute).

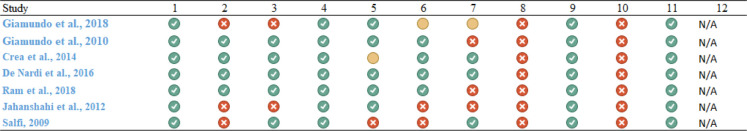	
Evaluated parameters
1	Was the study question or objective clearly stated?
2	Were eligibility/selection criteria for the study population prespecified and clearly described?
3	Were the participants in the study representative of those who would be eligible for the test/service/intervention in the general or clinical population of interest?
4	Were all eligible participants that met the prespecified entry criteria enrolled?
5	Was the sample size sufficiently large to provide confidence in the findings?
6	Was the test/service/intervention clearly described and delivered consistently across the study population?
7	Were the outcome measures prespecified, clearly defined, valid, reliable, and assessed consistently across all study participants?
8	Were the people assessing the outcomes blinded to the participants’ exposures/interventions?
9	Was the loss to follow-up after baseline 20% or less? Were those lost to follow-up accounted for in the analysis?
10	Did the statistical methods examine changes in outcome measures from before to after the intervention? Were statistical tests done that provided *p* values for the pre-to-post changes?
11	Were outcome measures of interest taken multiple times before the intervention and multiple times after the intervention (i.e., did they use an interrupted time-series design)?
12	If the intervention was conducted at a group level (e.g., a whole hospital, a community, etc.) did the statistical analysis take into account the use of individual-level data to determine effects at the group level?

Based on the results, we considered 1 paper of good quality (21), 3 of medium (19, 22, 23) and 5 of low quality (13, 15, 20, 24–26).

From the 3 controlled trials, only Naderan et al. 2017 (21) proved to be effectively double blind and randomized; Giamundo et al. 2011 (19) didn't properly ensure investigator blindness and Maloku et al. 2014 (20) didn't provide any information about the control given over the groups, whether they were randomized or if the participants were “blinded”, both acquiring a substantial degree of bias from these blunders. When evaluating the 7 clinical trials, we faced with no blindness (Parameter 8) and no little to no comparison between the before-after status of the participants (Parameter 10) throughout the different studies. Since the intervention was applied at an individual patient level, the 12th parameter was coded as NA (not applicable). Apart from these bias inducing flaws, the parameter with the least positive mark was the 7th, attaining to the outcome measures, accentuating the same problem observed in the 3 randomized controlled trials of heterogenicity between studies in respect to variables extracted and units of expression of those same variables. Only 4 trials in total expressed an acceptable number of different variables in clinically relevant units of measure (21–23, 25).

### Results of individual studies

Table 6 represents a summary of the main outcomes extracted of the studies included. The prospective study by Crea et al. (23) presented with the largest proportion of number of patients with bleeding immediately after the laser procedure up to 1 month post-surgery (25/97) in contrast with the clinical trial by Salfi (25) (1/200). The analysis of the results concerning the early postoperative pain at 24h shows that almost 80% (49/62) of patients presents values of pain between 0 and 1 in Ram et al. (15), in contrast with Maloku et al. (20) where the pain between 0 and 1 is present only in 25% of patients. The maximum mean value of pain after 24 h of surgery until 2 weeks after surgery is 1,4 in Giamundo et al. (19). The percentage of patients with pain until 12 months of follow-up in the different studies mostly ranged between 13.2% and 16.7%. However, Salfi (25) shows 0% of patients with pain until 12 months of follow-up. Naderan et al. (21) and Jahanshahi et al. (26) present a larger time until discharge compared to the other studies (24 and 18 h, respectively). Naderan et al. (21) was the study with the smallest percentage of patients with complete resolution of symptoms (70%), while in the other reports this value ranged between 86.3% and 95%. The percentage of patients with hemorrhoid recurrence did not exceed 9.7%.

**Table 6:** Main outcomes of the selected studies.

Summary of the main outcomes extracted of the studies included for systematic reviewing								
Study	Bleeding^a^	Early post operative pain (VAS)			Pain^b^	Time until discharge (h)	Symptom resolution^c^	Recurrence (% at 12 months)
		First 24 h	1–3 days	3–14 days				
Giamundo et al., 2018	-	–	–	1.1 (0–5)	–	–	90.3%	9.7%
Giamundo et al., 2010	–	–	1.4 ± 1.71	-	13.2%	–	–	–
Giamundo et al., 2011	7/30	–	1.1 (0–2)^d^	0.8 (0–2)	–	–	–	–
Crea et al., 2014	25/97	–	–	-	13.4%	6	More than 90%	–
De Nardi et al., 2016	–	–	–	-	15.7%	6	86.3%	5.5% (5 m)
Ram et al., 2018	–	49/62 [0–1] 13/62 [2–7]	–	-	–	1.52 ± 0.34	–	–
Naderan et al., 2017	4/30	1.6 ± 1.5	–	-	16.7%	24	70%	–
Jahanshahi et al., 2012	–	–	–	-	–	18	–	0%
Maloku et al., 2014	2/241	5/20 [0–1]; 15/20 [2–5]	–	19/20 [0–1]; 1/20 [2–5]	–	–	–	–
Salfi, 2009	1/200	–	–	-	0	–	95%	9%

^a^ Number of patients with bleeding immediately after laser procedure up to 1—month post-surgery.

^b^ Maximum percentage of patients with pain until the 12th month of follow-up.

^c^ The resolution of symptoms at 12 months of follow-up, except in De Nardi et al., 2016, where the follow-up was 30 days.

^d^ 0—Range of pain using VAS.

## Discussion

Our work aimed to give a broad view over laser haemorrhoidplasty and its post-operative follow up consequences.

The HeLP technique is ineffective in resolving the prolapse, as can be evaluated in the studies of Giamundo and De Nardi (22, 24). For this reason, in case of important prolapses, Giamundo recently introduced the HeLPexx procedure.

Accounting for the results previously described, we can see that in the few trials that report information regarding the same issue there appears to be a pattern between most of the trials. The results from the trials are consensual in terms of the reduced mean time of surgery, which, when taking into account the direct approach to the intended arteries, may direct to possible advantages of this procedure over more traditional techniques.

By analyzing the “bleeding” and “pain” parameters, one can see that the data is somewhat concordant, revealing a tendency of producing low number of symptoms, albeit the small differences in follow-up and the procedure itself within the trials. This could contribute to a good patient response regarding the treatment. When comparing the response to the treatment in terms of early post-operative pain, for the few trials which reported data the same way, the results were consistent, reporting mean values which could translate to a better patient response. However, these symptoms may not derive from the procedure itself but from non-resolved hemorrhoidal issues, though the symptoms and the non-complete resolution of symptoms being both sporadic.

When comparing with the literature on the Milligan-Morgan procedure, the trials that we described seemed to have an overall shorter operative time (27–29), shorter times until discharge (29, 30), lower rates of post-operative pain related complaints (31), higher symptom control rates (32) and lower rates of major complication (our papers reported no major complications) (33).

However, the same literature reported lower bleeding related complaints (30) and lower recurrence at 1 year follow-up (31, 33) for the Milligan-Morgan procedure. This information should be confirmed with posterior investigation with comparative trials between these two techniques.

The fact that the studies which did not use the doppler assisted technique had higher times until discharge is something of interest and could potentially be something worth looking to in future research.

In short, all of the trials concur that the laser, partly because it's a minimally invasive technique, can only bring benefits in terms of resolution of symptoms and absence of major complications.

### Limitations

One of the major limitations that we found within our analysis of the different papers was the difference of reported outcomes and the uncategorized nature of the different comorbidities reported. In this review, the authors agreed to only report about bleeding and pain, as these were not only the main symptoms but also the only ones that were systematically reported through the different trials. This proved to be a limitation of our study, since many other symptoms could have gone unnoticed and escaped our analysis, biasing any conclusions made in this review.

The heterogeneity between studies where different papers presented the same variable with different units was another of our major limitations. For example, two different papers presented the results of bleeding after the laser procedure in different ways comparing with the remaining articles: Giamundo et al. 2018 (24) referred to the bleeding in the form of a “bleeding score” using VAS, on the other hand, Giamundo et al. 2010 (13) didn't mention the number of patients with bleeding immediately after laser procedure up to 1 month post-surgery, but only the number of patients with bleeding intraoperatively, invalidating the data collection and possible comparison with the others articles.

The comparison of the outcome variable pain was also difficult since the values of early post-operative pain using VAS in the different studies weren't obtained at the same time after the procedure and Ram et al. 2018 (15) and Maloku et al. 2014 (20) didn't present mean values of pain in VAS. The short follow-up at which these symptoms were evaluated may also contribute to biased results.

The unavailability of some articles found in our electronic search and the fact that no reference list research was preformed may also prove to be a limitation. Some relevant studies could have been missed and could enrich our systematic review. The publication bias may also have influenced our results.

Other potential bias sources could be the anaesthesia (general anesthesia is not needed although some articles mention the use of it, which could be impacting their results, especially when attaining to early post-operative pain values) and the fact that some papers considered patients with first- and fourth-degree haemorrhoids (could influence post-operative complications values). Treatment at grade IV is advisable only in fragile patients with major comorbidities to reduce bleeding, the HeLP alone does not correct prolapse. For this reason, the inclusion of patients with grade IV hemorrhoids may represent a bias in the analysis of complications and relapses.

Brusciano et al. show (34) that the postoperative pain score was extremely low, the presence of slightly signifcant peri-anal wounds, no special anal hygienic measures and low surgical time using HeLP. In this study, the 100% of their population came back to daily activity 2 days after surgery. At a mean follow-up period of 8.6 months, existed a recurrence rate of 0%. Thus, resulting in a negligible postoperative discomfort, HeLP could be considered a painless and minimal invasive technique in the treatment of hemorrhoids disease.

### Conclusion

In summary, the laser haemorrhoidplasty procedure revealed a high therapeutic potential and a high beneficial impact on recuperation from the haemorrhoidplasty procedure, considering the reduced number of post-operative complications and comorbidities, the high symptom resolution and the reduced recurrence.

However, the limitations found within the studies must be taken into account and are the main reason that made impossible to proceed with a meta-analysis. Future high-quality investigations, with randomized studies are needed to compare HeLP technique with other dearterialization techniques in patients with Grade II and III haemorrhoids, obtaining better categorized results and complications, with a longer follow-up period.

## Data Availability

The original contributions presented in the study are included in the article/Supplementary Material, further inquiries can be directed to the corresponding author/s.
